# Co-culture of monocytes and *zona fasciculata* adrenal cells: An *in vitro* model to study the immune-adrenal cross-talk

**DOI:** 10.1016/j.mce.2021.111195

**Published:** 2021-04-15

**Authors:** Daniel P. Fudulu, George Horn, Georgina Hazell, Anne-Marie Lefrançois-Martinez, Antoine Martinez, Gianni D. Angelini, Stafford L. Lightman, Francesca Spiga

**Affiliations:** a Bristol Medical School: Translational Health Sciences, University of Bristol, Bristol, BS1 3NY, United Kingdom; b Bristol Heart Institute, University of Bristol, 68 Horfield Rd, Bristol, BS2 8ED, United Kingdom; c Génétique Reproduction & Développement, CNRS UMR 6293, Inserm U1103, Université Clermont Auvergne, 63001, Clermont-Ferrand, France

**Keywords:** Adrenal cortex, Steroidogenesis, Inflammation, Glucocorticoids

## Abstract

The hypothalamic-pituitary-adrenal axis is the primary neuroendocrine system activated to re-establish homeostasis during periods of stress, including critical illness and major surgery. During critical illness, evidence suggests that locally induced inflammation of the adrenal gland could facilitate immune-adrenal cross-talk and, in turn, modulate cortisol secretion. It has been hypothesized that immune cells are necessary to mediate the effect of inflammatory stimuli on the steroidogenic pathway that has been observed *in vivo*. To test this hypothesis, we developed and characterized a trans-well co-culture model of THP1 (human monocytic cell)-derived macrophages and ATC7 murine *zona fasciculata* adrenocortical cells. We found that co-culture of ATC7 and THP1 cells results in a significant increase in the basal levels of IL-6 mRNA in ATC7 cells, and this effect was potentiated by treatment with LPS. Addition of LPS to co-cultures of ATC7 and THP1 significantly decreased the expression of key adrenal steroidogenic enzymes (including StAR and DAX-1), and this was also found in ATC7 cells treated with pro-inflammatory cytokines. Moreover, 24-h treatment with the synthetic glucocorticoid dexamethasone prevented the effects of LPS stimulation on IL-6, StAR and DAX-1 mRNA in ATC7 cells co-cultured with THP1 cells. Our data suggest that the expression of IL-6 and steroidogenic genes in response to LPS depends on the activation of intra-adrenal immune cells. Moreover, we also show that the effects of LPS can be modulated by glucocorticoids in a time- and dose-dependent manner with potential implications for clinical practice.

## Highlights

• We propose a co-culture model of adrenocortical cells and monocytes to investigate the immune-steroidogenic cross-talk.
• LPS stimulation of co-cultured ATC7-THP1 cells increases IL-6 mRNA and reduces StAR and DAX-1 mRNA expression.
• Cytokines effects on IL-6 and steroidogenic genes mRNA are different in co-cultured ATC7- THP1 cells and ATC7 cells alone.
• DEX prevents the LPS induced IL-6 mRNA expression and steroidogenic gene in ATC7-THP1 cells in a dose-dependent manner.
• ACTH induced adrenal IL-6 mRNA expression and steroidogenic genes activation are modulated by co-culture with THP1 cells.

## 1 Introduction

The acute stress response in man includes activation of the sympathetic nervous system, the hypothalamic-pituitary-adrenal (HPA) axis, as well as immunological and haematological responses (Desborough 2000). Internal and external stressors are integrated through the brain stem and limbic areas, projecting to the hypothalamic paraventricular nucleus, which innervates the median eminence to release CRH into the portal circulation and thence corticotroph cells of the anterior pituitary. These cells release ACTH into the systemic circulation which, in turn, activates both the production and release of glucocorticoids (corticosterone in rodents and predominantly cortisol in humans cortisol) from the *zona fasciculata* of the adrenal gland, which is vital for homeostatic regulation (Spiga et al., 2014).

In the adrenal cortex, ACTH binds to the melanocortin type-2 receptor (MC2R), leading to activation of the protein kinase A (PKA) pathway, which in turn results in activation of steroidogenic gene expression, via non-genomic and genomic pathways. (reviewed in (Miller and Auchus 2011)). While the non-genomic pathway includes the phosphorylation and activation, of steroidogenic proteins including the rate-limiting steroidogenic acute regulatory protein (StAR) (Stocco and Clark 1996; Arakane et al., 1997; Spiga et al., 2017), the genomic pathway regulates the transcription of steroidogenic proteins and its transcriptional regulators. This includes transcription of steroidogenic proteins such as StAR, MC2R, melanocortin receptor accessory protein (MRAP, a protein that regulates MC2R expression (Metherell et al., 2005) as well as the orphan nuclear receptor – steroidogenic factor (SF-1) (Sugawara et al., 1996) and the transcriptional inhibitor DAX-1 (the dosage-sensitive sex reversal, adrenal hypoplasia congenital critical region on the X chromosome, gene 1) (Zazopoulos et al., 1997). Within the HPA axis, glucocorticoid secretion is regulated by a negative feedback mechanisms wherbay cortisol exert inhibitory effects at the pituitary and hypothalamic levels.

According to this “traditional” model, any increase in ACTH secretion in response to acute stress will result in concomitant increased cortisol. However, during surgery and critical illness, a so-called “ACTH-cortisol dissociation” occurs (Gibbison and Spiga 2014; Boonen et al., 2015). Systemic administration of LPS in the rat results in a similar pattern: an initial rise in ACTH and corticosterone, followed by a return of ACTH to basal levels within 6 h while the corticosterone remained elevated for a further 4 h (Gibbison and Spiga 2014). The mechanisms behind these findings are a matter of debate and studies have suggested altered cortisol metabolism (Boonen et al., 2013), increased sensitivity of the adrenal cortex to ACTH (Gibbison et al., 2015) and local, adrenal “tissue” mechanisms that could involve the cellular interaction between the adrenal cells and the surrounding immune cells (Boonen et al., 2015). This cross-talk can occur via cytokines produced by adrenal cells themselves or by the neighbouring immune cells to regulate steroidogenesis (Bornstein et al., 2004a). The above hypotheses are supported by several studies. Lipopolysaccharide-induced systemic inflammation is accompanied by infiltration of leukocytes in the adrenal gland of rats (Kanczkowski et al., 2013a). Furthermore, in a mice model of sepsis-induced by caecal ligation and puncture, the non-survivor mice have a significant increase of interleukin-6 (IL-6), interleukin-1β (IL-1β), and tumour necrosis factor-α (TNFα) in adrenal protein extracts (Jennewein et al., 2016). Local modulation of the adrenocortical cell function can also occur directly, for example, via the toll-like receptor (TLR), or via circulating cytokines activating cytokine receptors, both of which are expressed in adrenocortical cells (Bornstein et al., 2004b). However, it remains unclear if the plasma level of immune-derived cytokines is high enough to regulate the adrenocortical steroidogenesis directly or if they have to be secreted locally within the adrenal gland (Ehrhart-Bornstein et al., 1998). Adrenal cells do produce a variety of cytokines such as IL-1, interferon-gamma inducing factor (IGIF), IL-6 and TNFα (Judd 1997; Bornstein et al., 2004a), and steroidogenesis is influenced directly by IL-1α, IL-1β, interleukin IL-2, IL-6, TNFα, interferon-alpha (IFNα) both *in vitro* (Ehrhart-Bornstein et al., 1998) and *in vivo* (Spiga et al., 2020). Furthermore, a previous study using primary cultures of human adrenocortical cells co-cultured with human monocytes has shown a significant increase in cortisol production by the adrenal cells. In this study, the monocyte induced cortisol increase was much higher than that resulting from IL-1 treatment alone (Whitcomb et al., 1988).

The current study describes a novel co-culture model of adrenocortical tumour cell lines murine ATC7 cells, with complete *zona fasciculata* (ZF) cell phenotype (Ragazzon et al., 2006; Hazell et al., 2019) and macrophages derived from THP1 monocytes (a human monocytic cell line derived from an acute monocytic leukaemia patient) (Tsuchiya et al., 1980). Using this model, we explored the effects of an immunological stimulus (lipopolysaccharide, LPS) on the expression of the pro-inflammatory cytokine IL-6 as well as the expression of genes involved in steroidogenesis in ATC7 cells, both in basal conditions and under ACTH stimulation. Since synthetic glucocorticoids are still widely used in clinical practice to modulate the immune and adrenal response to acute stress observed during sepsis or surgery and their efficacy and mechanism of action remain a matter of intense debate (Fudulu et al., 2018), we also investigated the temporal effects of glucocorticoid treatment on the ATC7 cell responses to LPS.

## 2 Material and methods

### 2.1 Single-cell type culture, trans-well co-culture and cell treatments

**Fig. 1:**
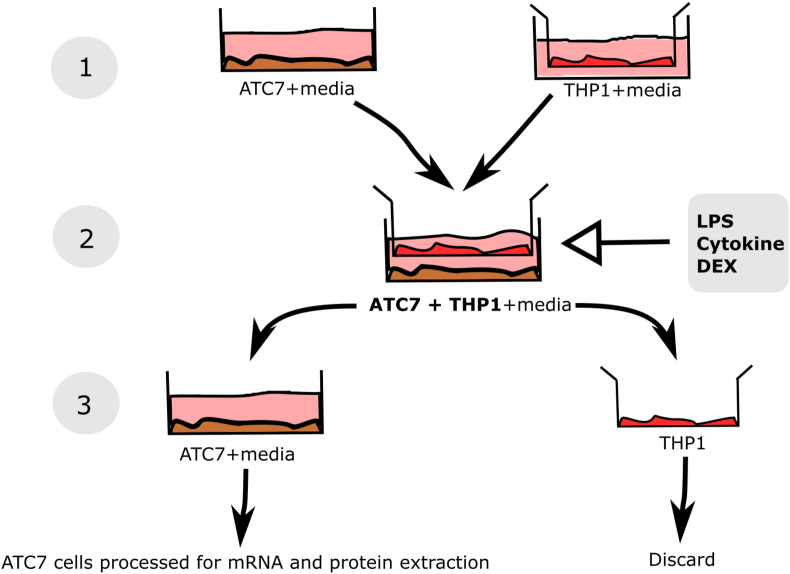
**Diagram of methods used to co-culture ATC7 and THP1 cells.** The ATC7 cells and the THP1 cells are first cultured separately (1). Then cells are co-incubated using a transwell system and undergo various treatments with LPS, DEX or cytokines (2). At the end of treatment, the well containing the THP1 cells is removed and cells are discarded. ATC7 cells are collected and processed for mRNA and protein extraction and measurement by RT-qPCR and western immunoblotting, respectively (3).

Murine adrenocortical tumour ATC7 cells (a kind gift from Dr Pierre Val, Université Clermont Auvergne, Clermont-Ferrand, France), were cultured as previously described (Hazell et al., 2019). Human monocytic THP1 cells were purchased from Sigma (Sigma, Gillingham, UK). The reason behind the use of human macrophagic cells is that the difference in the species in the two cell types would allow us to measure specifically adrenal (murine) or macrophagic (human) cytokines expression. The methods of the co-culture experiments are summarised in Fig. 1. THP1 cells were cultured in suspension in 75 cm^2^ tissue-culture flasks in DMEM at 37 °C in a 5%CO_2_-95% air atmosphere. The medium was supplemented with 20% horse serum penicillin (100U/ml) and streptomycin (100 μg/ml). Cells were passaged every 3–4 days, and culture media changed every two days. Differentiation of THP1 cells was achieved by resuspending THP1 cells in medium containing 100 nM PMA (Sigma) in 6 wells plate polycarbonate cell culture inserts (TC inserts, Sarsted, Nümbrecht, Germany). Cells were left to differentiate for 72 h then washed twice with 1x PBS (phosphate-buffered saline, pH 7.4, ThermoFisher, Waltham, MA USA). The insert containing THP1 cells was then transferred into a six wells plate containing ATC7 cells and incubated in serum-free media (DMEM/F12/0.1% BSA). The ratio of ATC7 cells co-incubated with THP1 cells was kept at 1:2 for all experiments except on the ratio experiment in which different ratio ATC7: THP1 were tested. Both ATC7 and THP1 cells were serum-starved in serum-free medium supplemented with 0.1% BSA approximately 16–24 h before the start of each experiment. ATC7 and/or ATC7-THP1 cells were treated with: LPS (Lipopolysaccharides from *Escherichia coli* O111: B4; Sigma, UK), pro-inflammatory cytokines (mouse IL-1β, IL-6 and TNFα, 10 nM/mL; Miltenyi Biotec GmbH, Bergisch Gladbach, Germany), dexamethasone (DEX, Dexamethasone 21-phosphate disodium salt; Sigma); ACTH (adrenocorticotropic hormone from porcine pituitary, Fragment 1–39; Sigma) as described in detail for each experiment in the result section. At the end of each experiment, cells were washed with ice-cold phosphate-buffered saline (PBS), and then sodium dodecyl sulfate (SDS)-lysis buffer (2% SDS, 50 mM Tris pH 6.8, 10% glycerol) was added to each well. Cells were scraped off, and the lysate was collected in two aliquots and stored at −20C until processing for RNA and protein extraction as described in Fig. 1.

### 2.2 Quantitative RT-PCR

For RNA quantification cells were lysed in RNA lysis buffer, and total RNA was purified using Ambion Pure-Link kit (Invitrogen, ThermoFisher Scientific). The cDNA template was reverse-transcribed from 1000 ng of total RNA using Cloned AMV First-Strand cDNA synthesis kit (Invitrogen, ThermoFisher Scientific). RTqPCR was performed as previously described (Park et al., 2013) using Power SYBR green PCR mix (Applied Biosystems, ThermoFisher Scientific) and 4 ng cDNA template. RTqPCR primers (listed in Supplementary Table 1) were used at a final concentration of 200 nM and designed to span an exonic-exonic region to detect mature transcript (mRNA). Each sample was analised in duplicate and GAPDH was used as a house-keeping gene.

### 2.3 Western immunoblotting

For protein quantification cells were lysed in SDS lysis buffer (2% SDS; 50 mM Tris pH 6.8; 10% glycerol) and Western immunoblotting performed as described in (Hazell et al., 2019). In brief, all membranes were blocked with 1% BSA in Tris-buffered saline/0.05% Tween 20 (TBS/T) and probed with primary rabbit antibodies directed to StAR (1:1000; Santa Cruz Biotechnology, USA), pCREB (1:1000; Cell Signalling Technology, Inc., USA), followed by horseradish peroxidase-conjugated donkey α-rabbit secondary antibody (1:5000; Santa Cruz Biotechnology). Vinculin (Goat α-vinculin primary (1:5000) followed by a Donkey α-Goat secondary (1:5000) (both Santa Cruz Biotechnology) was used as a loading control as previously shown (Hazell et al., 2019). Protein bands were visualized with Luminata Forte Western HRP substrate (Millipore Corporation, Billerica, MA, USA) using a G BOX (Syngene, Cambridge, UK) and densitometry was determined using Image J (developed at the National Institutes of Health and freely available at http://rsb.info.nih.gov).

### 2.4 Statistic

Graph Pad Prism version 7.00 (Graph Pad Software, La Jolla, CA, USA) and SPSS version 24 (IBM Corp., Armonk, NY, USA) was used for data graphing and statistical analysis, respectively. All data are expressed as mean ± SEM. For all experiments, one-way, two-way or three-way analysis of variance (ANOVA) was used. When a significant effect of main factors or interactions was found, a Tukey's multiple comparison test (post one-way and two-way ANOVA) or Fisher's LSD post hoc test (post three-way ANOVA) was used. Significance was set at P ≤ 0.05.

### 2.5 Results

#### 2.5.1 LPS stimulation of ATC7 cells co-cultured with THP1 cells induces the expression of adrenal IL-6 mRNA

**Fig. 2:**
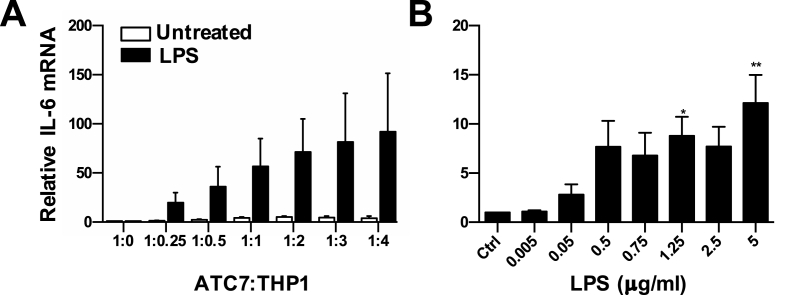
**Effect of LPS treatment in ATC7 cells co-cultured with THP1 cells on IL-6 mRNA expression in ATC7 cells.** ATC7 cells were cultured alone (**A**) or co-cultured with THP1 cells (**A** and **B**) and treated with LPS for 24-h. Relative levels of IL-6 mRNA were measured in ATC7 cells by RTqPCR and GAPDH was used as a house-keeping gene. (**A**) Effect of increasing ATC7:THP1 cells ratio and LPS treatment (5 μg/mL) on IL-6 mRNA expression in ATC7 cells. Data are the mean ± SEM (n = 4/group) and are expressed as fold induction of untreated ATC7 (1: 0) cells. Data were analyzed by two-way ANOVA, followed by Tukey's multiple comparison test. (**B**) Effect of increasing doses of LPS in ATC7 cells co-cultured with THP1 cells (1:2 ratio) on IL-6 mRNA expression in ATC7 cells. Data are the mean ± SEM (n = 4/group) and are expressed as fold induction of untreated ATC7 cells co-cultured with THP1 cells (Ctrl). Data were analyzed by one-way ANOVA, followed by Tukey's multiple comparison test. Effect of LPS: *P < 0.05; **P < 0.01 *vs* Ctrl.

Our preliminary experiments demonstrated no significant changes in the expression of IL-6 mRNA in ATC7 cells in response to LPS stimulation, either alone (Supplementary Fig. 1) or in co-treatment with Interferon-gamma (Supplementary Fig. 2). Because resident macrophages are found in basal unstimulated conditions in the adrenal cortex *in vivo* (Boonen et al., 2015), we hypothesized that ATC7 cells would require the presence of activated immune cells for LPS to be able to affect the expression of pro-inflammatory cytokines and steroidogenic genes. Therefore, in this experiment we tested the effect of co-culturing ATC7 cells with of THP1 derived macrophages (referred to as THP1) cells at various ratios, as well as the effect of treatment with various doses of LPS for 24 h (Fig. 2). Two-way ANOVA showed a significant effect of LPS (P < 0.0003) but no effect of THP1 co-culture, nor interaction, was observed on IL-6 mRNA (Fig. 2A). Although higher levels of IL-6 mRNA could be observed in co-cultured ATC7 cells co-cultured with THP1 cells treated with LPS, post hoc test did not detect any specific difference between experimental groups. Next, we evaluated the dose-response effect of 24-h LPS stimulation on ATC7 cells co-cultured with THP1 cells (co-cultured at a 1 ATC7: 2 THP1 cells ratio. One-way ANOVA revealed a significant effect of LPS on IL-6 mRNA expression (P = 0.0032; Fig. 2B), with a significant increase observed in cells treated with LPS at the dose of 1.25 μg/mL and 5 μg/mL concentration (P = 0.0453 and P = 0.0024, respectively). In summary, we demonstrate the co-culture of ATC7 cells with THP1 cells increases the IL-6 mRNA expression. This increase is significantly potentiated by LPS stimulation in a dose-dependent manner.

#### 2.5.2 Effect of increasing ratio of THP1 co-culture and LPS stimulation on steroidogenic gene expression in ATC7 cells

**Fig. 3:**
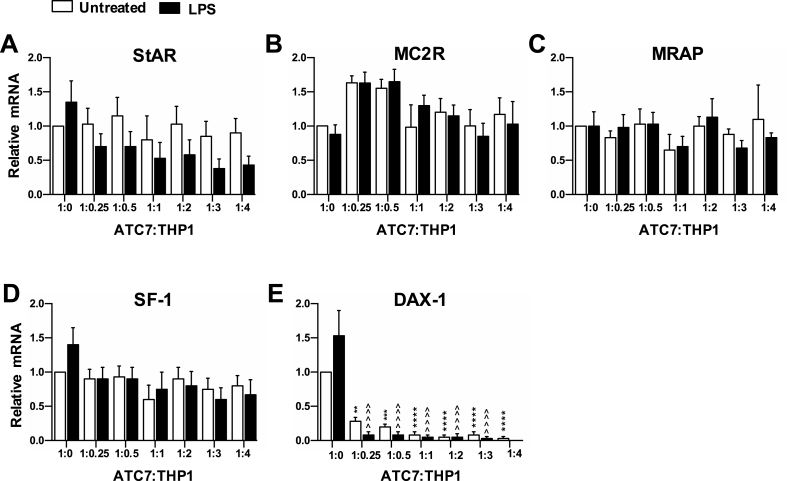
**Effect of increasing THP1 cells ratio and LPS stimulation on steroidogenic gene expression in ATC7 cells.** ACT7 cells were cultured alone or co-cultured with THP1 cells and treated with LPS (5 μg/mL) for 24-h. The relative level of steroidogenic genes mRNA was measured in ATC7 cells by RTqPCR and GAPDH was used as a house-keeping gene. Data are the mean ± SEM (n = 4/group) and are expressed as fold induction of untreated ATC7 cells (1:0); data were analyzed by two-way ANOVA followed by Tukey's multiple comparison test. Effect of LPS: **P < 0.01; ***P < 0.001; ****P < 0.0001 *vs* untreated ATC7 (1:0) cells; ^^^^ P < 0.0001 *vs* LPS-treated ATC7 (1:0) cells.

Significant effects of ACT7-THP1 cells co-culture and LPS treatment were also found on the expression of key steroidogenic genes (Fig. 3). Specifically, there was an overall effect of LPS on StAR mRNA (P = 0.021; Fig. 3A) and an overall effect of THP1 co-culture on MC2R mRNA levels (P = 0.001; Fig. 3B). As observed for IL-6 mRNA, post hoc analysis did not reveal any significant differences between groups; however, StAR mRNA levels appeared reduced in LPS-treated ATC7 cells co-cultured with THP1 cells, compared to untreated ATC7 cells co-cultured with THP1 cells, and MC2R mRNA levels were elevated in ACT7-THP1 cells with low THP1 ratio (0.25 and 0.5), compared to single ATC7 cells. No effects of co-culture, or of LPS, were found on MRAP mRNA (Fig. 3C) or SF-1 mRNA (Fig. 3D). However, a significant effect of THP1 (P < 0.0001), as well as a significant effect of THP1xLPS interaction (P = 0.048), was found on DAX-1 mRNA (Fig. 3E). The post hoc test revealed a significant decrease of DAX-1 mRNA in ATC7 cells co-cultured with THP1 cells treated with either LPS or vehicle. Interestingly, in ATC7 only cells, there was a trend of increase in the expression of DAX-1 mRNA in response to LPS stimulation (P = 0.072) compared to ATC7 cells treated with vehicle. In summary, in this experiment, we demonstrate that LPS stimulation of ATC7 cells co-cultured with THP1 cells modulates the steroidogenic pathway mainly by reducing both StAR mRNA expression and DAX-1 mRNA expression.

#### 2.5.3 Dose-dependent effects of LPS on the expression of steroidogenic genes in ATC7 cells co-cultured with THP1 cells

**Fig. 4:**
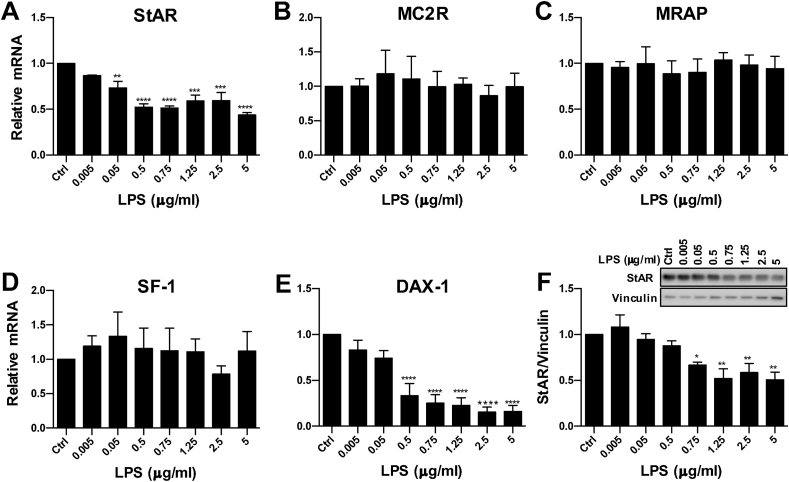
**Effect of LPS treatment in ATC7 cells co-cultured with THP1 cells on steroidogenic gene expression and StAR protein in ATC7 cells.** ATC7 cells were co-cultured with THP1 at 1:2 ratio and treated with LPS for 24-h. (**A-E**) Effect of increasing doses of LPS on steroidogenic genes mRNA expression. Relative levels of IL-6 and steroidogenic genes mRNA were measured in ATC7 cells by RTqPCR and GAPDH was used as a house-keeping gene. (**F**) Effect of increasing doses LPS on StAR protein in ATC7 cells. Relative levels of StAR protein were measured in ATC7 cells by western immunoblotting, and data were normalized to vinculin. Data are the mean ± SEM (n = 4/group) and are expressed as fold induction of untreated ATC7 cells co-cultured with THP1 cells (Ctrl); data were analyzed by one-way ANOVA followed by Tukey's multiple comparison test. *P < 0.05; **P < 0.01; ***P < 0.001; ****P < 0.0001 *vs* Ctrl.

In this experiment, we evaluated the dose-response effect of 24-h LPS stimulation on the expression of steroidogenic genes in ATC7 cells co-cultured with THP1 cells at a 1:2 cells ratio (Fig. 4). Two-Way ANOVA revealed a significant effect of LPS on StAR mRNA (P < 0.0001; Fig. 4A) and DAX-1 mRNA (P < 0.0001; Fig. 4E). Compared to controls, StAR mRNA expression was significantly decreased in cells treated with LPS at doses between 0.05 and 5 μg/mL, (p < 0.0001; Fig. 4A), whereas a significant decrease in DAX-1 was observed in cells treated with LPS at doses between 0.5 and 5 μg/mL (Fig. 4E). Consistent with the previous experiment, there was no effect of LPS on MC2R, MRAP and SF-1 mRNA (Fig. 3B–D). In accordance with the mRNA data, analysis of StAR protein showed a significant effect of LPS (P < 0.0001; Fig. 4F), with a significant decrease in cells treated with LPS doses between 0.75 and 5 μg/mL. In summary, data from this experiment demonstrate that the LPS induced suppression of StAR mRNA expression and protein translation and DAX-1 mRNA expression occurs in a dose-dependent manner.

#### 2.5.4 Time-course effect of cytokines on IL-6 and steroidogenic genes mRNA levels in ATC7 only cells and in ATC7 cells co-cultured with THP1 cells

**Fig. 5:**
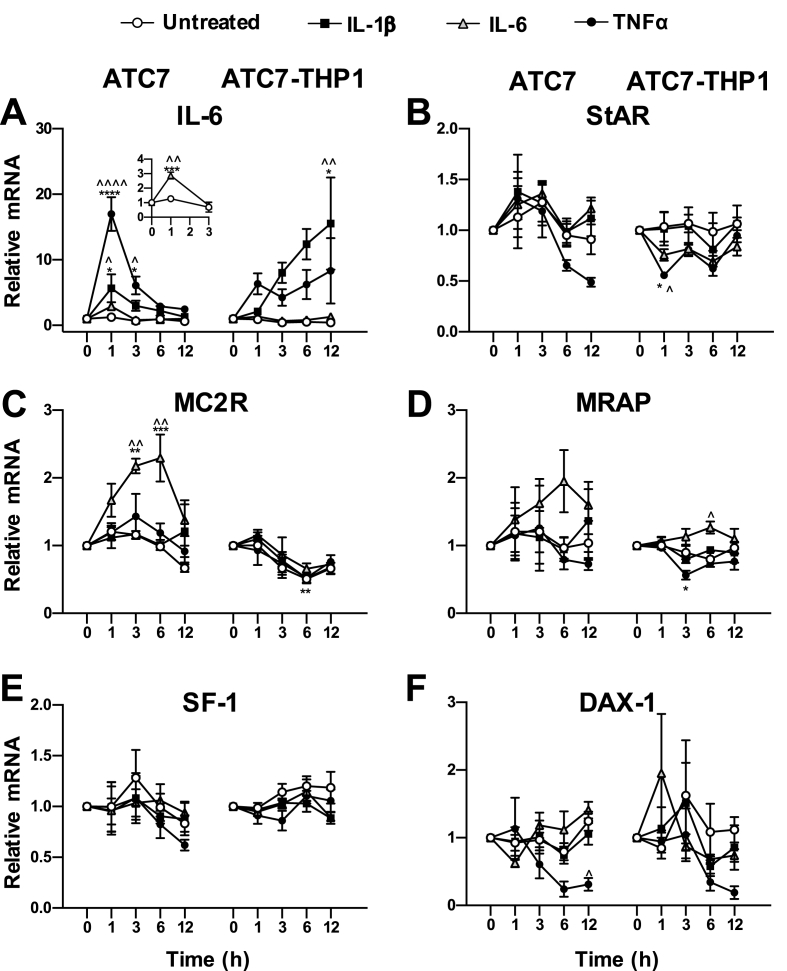
**Effect of cytokines treatment in ATC7 only cells and in ATC7 cells co-cultured with THP1 cells on IL-6 mRNA and steroidogenic genes mRNA expression in ATC7 cells**. ATC7 only cells and ATC7 cells co-cultured with THP1 cells were either left untreated or treated with IL-1β (10 ng/mL), IL-6 (10 ng/mL) or TNFα (10 ng/mL) for 1 h, 3 h, 6 h and 12 h. Relative levels of IL-6 and steroidogenic genes mRNA were measured in ATC7 cells by RTqPCR and GAPDH was used as house-keeping gene. Data are the mean ± SEM (n = 3/group). and are expressed as fold induction of untreated ATC7 only cells or ATC7 cells co-cultured with THP1 cells at time 0 (Ctrl); data were analyzed by two-way ANOVA followed by Tukey's multiple comparison test. *P < 0.05; **P < 0.01 *vs* Ctrl; ^P < 0.05; ^^P < 0.01 *vs* untreated cells at the same time point.

Treatment of THP1 cells with LPS results in the secretion of pro-inflammatory cytokines, including IL-1β, IL-6 and TNFα (Wehrhahn et al., 2010; Palacio *et al.* 2011; Schildberger et al., 2013a). Therefore, to investigate whether the effects of LPS on IL-6 mRNA and on steroidogenic gene mRNA may be mediated by specific macrophage's cytokines, in this experiment we tested the time-course of the effects of IL-1β, IL-6 and TNFα treatment in ATC7 only cells and in ATC7 cells co-cultured with THP1 cells (Fig. 5).

The effect of cytokines on IL-6 mRNA is shown in Fig. 5A. In ATC7 only cells, we found a significant effect of IL-1β treatment (P = 0.003), time (P = 0.030) and interaction (P = 0.089), with a significant increase in IL-6 mRNA at 1 h (P = 0.013 *vs* time 0; P = 0.023 *vs* untreated 1 h); we also found a significant effect of IL-6 treatment (P = 0.012), time (P = 0.0001) and interaction (P = 0.014), with a significant increase in IL-6 mRNA at 1 h (P = 0.001 *vs* time 0; P = 0.004 *vs* untreated 1 h, see insert in Fig. 5A), and a significant effect of TNFα treatment, time and interaction (all P < 0.0001) with an increase in IL-6 mRNA at 1 h (P < 0.0001 *vs* time 0 and *vs* untreated 1 h) and at 3 h (P = 0.027 *vs* time 0; P = 0.016 *vs* untreated 3 h). In ATC7-THP1 cells, we found a significant effect of IL-1β treatment (P = 0.0001), time (P = 0.035) and interaction (P = 0.020), with a significant increase in IL-6 mRNA at 12 h (P = 0.010 *vs* time 0; P = 0.007 *vs* untreated 12 h), but only a trend of increase at 6 h (P = 0.072 *vs* time 0; P = 0.052 *vs* untreated 6 h). We also found a significant effect of IL-6 treatment (P = 0.006) and time (P = 0.045), with a trend at 12 h (P = 0.096 *vs* untreated at 12 h), and an effect of TNFα treatment in cells treated with (P = 0.001), but no significant changes in the post hoc test.

The effect of cytokines on StAR mRNA is shown in Fig. 5B. In ATC7 only cells, we found no effect of IL-1β, IL-6 or TNFα treatment nor effect of time in cells treated with IL-1β or IL-6, but a significant effect of time in cells treated with TNFα (P = 0.014), with no significant changes found in the post hoc analysis. In ATC7-THP1 cells, we found no effect of IL-1β treatment or time, whereas there was an effect of IL-6 treatment (P = 0.002) but no significant changes in the post hoc analysis. We also found an effect of TNFα treatment (P = 0.0003), but only a trend of the effect of time (P = 0.0541) and interactions (P = 0.081); post hoc test revealed a significant decrease in StAR at 1 h (P = 0.043 *vs* time 0; P = 0.023 *vs* untreated 1 h).

The effect of cytokines on MC2R mRNA is shown in Fig. 5C. In ATC7 only cells, we found no effect of IL-1β treatment or time, whereas there was a significant effect of IL-6 treatment (P < 0.0001), time (P = 0.0006) and interaction (P = 0.008), with a significant increase in MC2R mRNA at 3 h (P = 0.002 *vs* time 0; P = 0.008 *vs* untreated 3 h) and at 6 h (P = 0.001 *vs* time 0; P = 0.001 *vs* untreated 6 h). We also found a significant effect of time in cells treated with TNFα (P = 0.011), but no significant changes in the post hoc analysis. In ATC7-THP1 cells, we found an effect of time in cells treated with IL-1β (P < 0.0001), IL-6 (P = 0.0003) or TNFα (P = 0.002), and a trend of the effect of IL-6 treatment (P = 0.0562), with a decrease in MC2R mRNA at 6 h in both untreated cells (P = 0.007 *vs* time 0) and cells treated with IL-1β (P = 0.008 *vs* time 0).

The effect of cytokines on MRAP mRNA is shown in Fig. 5D. In ATC7 only cells, we found no effect of treatment or time in cells treated with IL-1β or TNFα, but a significant effect of IL-6 treatment (P = 0.04), with no significant changes in the post hoc analysis. In ATC7-THP1 cells, we found no effect of IL-1β treatment or time, but a significant effect of IL-6 treatment (P = 0.005), with a significant increase in MRAP mRNA at 6 h (P = 0.04 *vs* untreated 6 h). We also found a significant effect of TNFα treatment (P = 0.003) and time (P = 0.012), with a significant decrease in MRAP mRNA at 3 h (P = 0.011 *vs* time 0; P = 0.087 *vs* untreated 6 h).

The effect of cytokines on SF-1 mRNA is shown in Fig. 5E. In ATC7 only cells, we found no effect of treatment or time in cells treated with IL-1β, IL-6 or TNFα. In contrast, in ATC7-THP1 cells, we found a significant effect of IL-1β treatment (P = 0.024) and TNFα treatment (P = 0.042), but only a trend of the effect of IL-6 treatment (P = 0.055), with no significant changes in the post hoc analysis for any of the cytokines treatments group.

The effect of cytokines on DAX-1 mRNA is shown in Fig. 5F. In ATC7 only cells, we found no effect of IL-1β treatment or time, whereas we found a significant effect of time in cells treated with IL-6, with no significant changes in the post hoc analysis, and a significant effect of TNFα treatment (P = 0.011) and interaction (P = 0.045), and a trend of effect in time (P = 0.076), with a decrease in DAX-1 mRNA at 12 h (P = 0.048). In ATC7-THP1 cells, we found no effect of treatment or time in cells treated with IL-1β or IL-6, and only a trend of the effect of TNFα (P = 0.054). In summary, cytokines treatment can affect the levels of IL-6 and steroidogenic genes expression, and these effects are different in ATC7 cells co-cultured with THP1 and ATC7 alone.

#### 2.5.5 Effects of dexamethasone and LPS co-treatment on IL-6 and steroidogenic gene mRNA levels in ATC7 cells co-cultured with THP1 cells

**Fig. 6:**
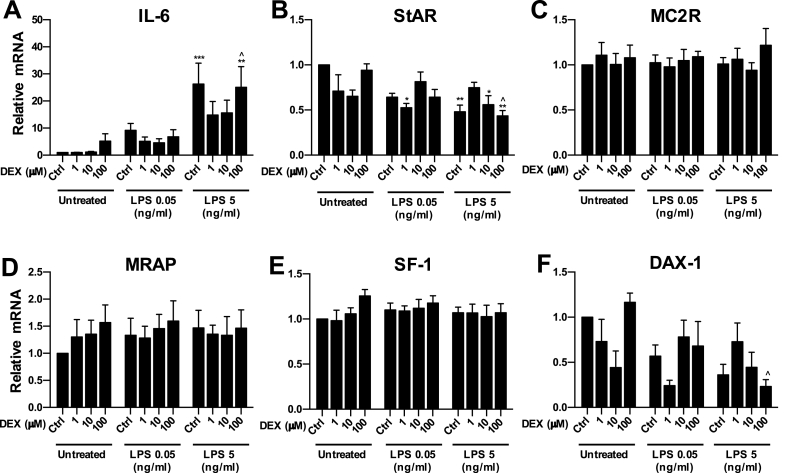
**Effect of dexamethasone and LPS treatment in ATC7 cells co-cultured with THP1 cells on IL-6 and steroidogenic genes mRNA expression ATC7 cells.** ATC7 cells co-cultured with THP1 cells were treated with dexamethasone (DEX, 1, 10 and 100 μM) and/or LPS (0.05 and 5 μg/mL) for 24-h. Relative levels of Il-6 and steroidogenic genes mRNA were measured in ATC7 cells by RTqPCR and GAPDH was used as a house-keeping gene. Data are the mean ± SEM (n = 6/group) and are expressed as fold induction of untreated Ctrl cells; data were analyzed by two-way ANOVA followed by Tukey's multiple comparison test. *P < 0.05; **P < 0.01; ***P < 0.001 *vs* untreated Ctrl; ^P < 0.05 *vs* untreated cells of the same DEX treatment group.

In the following sets of experiments, we tested the hypothesis that the effects of LPS on IL-6 and steroidogenic gene expression can be modulated by treatment with the synthetic glucocorticoid dexamethasone (DEX). Firstly, we investigated the effect of 24-h co-treatment with DEX and LPS on the expression of IL-6 mRNA and steroidogenic genes mRNA in ATC7 cells co-cultured with THP1 cells (Fig. 6). We found a significant overall effect of LPS (P < 0.0001) on IL-6 mRNA, but no significant effect of DEX nor interaction (Fig. 6A). Post hoc test revealed a significant increase in IL-6 mRNA in control cells treated with 5 μg/mL LPS (P = 0.0009) and in cells co-treated with 5 μg/mL LPS and 100 μM DEX (P = 0.0037), compared to untreated control and 100 μM DEX-treated ATC7-THP1 cells, respectively; the effect of 5 μg/mL LPS was not observed in ATC7-THP1 cells co-treated with 1 μM and 10 μM DEX. We did not observe any effect of the lower dose of LPS (0.05 μg/mL LPS) neither in control nor in DEX-treated ATC7-THP1 cells.

Analysis of the effects of DEX and LPS on steroidogenic gene expression revealed a significant effect of DEX (P < 0.0001) and a significant DEX × LPS interaction (P = 0.0009) on StAR mRNA (Fig. 6B). StAR mRNA levels were decreased in cells treated with 5 μg/mL LPS (P = 0.0037), compared to control ATC7-THP1 cells, and these effects were prevented by 1 μM DEX, but not by 10 and 100 μM DEX. We also observed a significant decrease in StAR mRNA in cells treated with both 1 μM DEX and 0.05 μg/mL LPS (P = 0.0116) compared to control ATC7-THP1 cells, suggesting a synergistic effect of DEX and LPS at low doses. There was also a significant effect of DEX (P = 0.0048) and DEX × LPS interaction (P = 0.0051) on DAX-1 mRNA (Fig. 6F). However, post hoc analysis did not reveal any significant effect of LPS or DEX alone, but a trend of decrease in DAX-1 mRNA levels was found in cells treated with 1 μM DEX and 0.05 μg/mL LPS and in cells treated with 100 μM DEX and 5 μg/mL LPS (P = 0.0887 and P = 0.0800, respectively, compared to control ATC7-THP1 cells). Co-treatment with DEX and LPS did not affect MC2R, MRAP or SF-1 mRNA levels (Fig. 6C–E). In this experiment, we show that glucocorticoid co-administration can prevent the LPS induced IL-6 mRNA expression and steroidogenic gene changes (StAR mRNA and DAX-1 mRNA expression) in ATC7-THP1 cells in a dose-dependent manner.

#### 2.5.6 Effects of dexamethasone pre-treatment on LPS-induced changes in IL-6 and steroidogenic gene mRNA levels in ATC7 cells co-cultured with THP1 cells

**Fig. 7:**
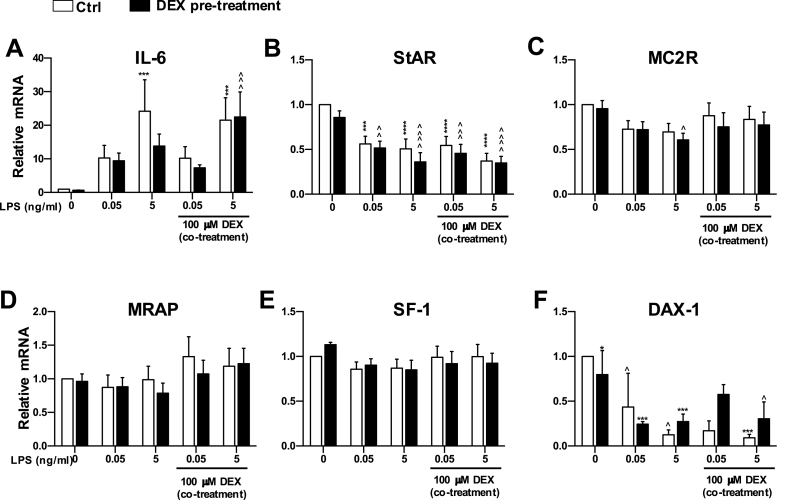
**Effect dexamethasone pre-treatment, and dexamethasone and LPS co-treatment in ATC7 cells co-cultured with THP1 cells on IL-6 and steroidogenic genes mRNA expression in ATC7 cells.** ATC7 cells co-cultured with THP1 cells were pre-treated with dexamethasone (DEX, 100 μM) for 24-h, and then co-treated with LPS (0.05 or 5 μg/mL) and/or dexamethasone (100 μM) for 24-h. Relative levels of IL-6 and steroidogenic genes mRNA were measured in ATC7 cells by RTqPCR and GAPDH was used as house-keeping gene. Data are the mean ± SEM (n = 6/group) and are expressed as fold induction of untreated Ctrl; data were analyzed by three-way ANOVA followed by Fisher's LSD post hoc test. ***P < 0.001; ****P < 0.0001 *vs* LPS-untreated Ctrl; ^P < 0.05; ^^P < 0.01 ^^^P < 0.001; ^^^^P < 0.0001 *vs* Ctrl cells of the same LPS ± DEX treatment. The closed bars denote DEX pre-treated cells.

Our previous experiment has shown that ATC7 cells co-cultured with THP1 cells treated with DEX prevent some of the effects of LPS on IL-6, StAR and DAX-1 mRNA, but only at the lower doses of 1 μM and 10 μM. In this experiment, we aimed to test whether pre-treatment with 100 nM DEX was able to prevent LPS-induced effects on gene transcription in ATC7 cells co-cultured with THP1 cells. Twenty-four hours treatment with DEX was followed by 24-h treatment with LPS (at the dose of 0.05 of 5 μg/mL) alone or in combination with DEX (Fig. 7). Three-way ANOVA revealed a significant effect of LPS on IL-6 mRNA (P < 0.00001) but no effect of DEX pre-treatment, DEX co-treatment, nor interactions (Fig. 7A). IL-6 mRNA levels were significantly higher in cells treated with 5 nM LPS, and this effect was prevented in cells pre-treated with DEX, but not in cells both pre- and co-treated with DEX. Three-way ANOVA also revealed a significant effect of LPS on StAR (P < 0.0001; Fig. 7B) and DAX-1 mRNA (P < 0.0001; Fig. 7F), but no effect of DEX pre-treatment, DEX co-treatment, nor interactions on either gene. LPS treatment decreased StAR mRNA levels and neither pre- nor co-treatment with DEX prevented these effects. Similarly, LPS treatment decreased DAX-1 mRNA levels, but this effect was prevented in cells treated with 0.05 μg/mL LPS pre- and co-treated with DEX. We also observed an overall effect of LPS on MC2R mRNA (P = 0.0180; Fig. 7C); however, a significant decrease in MC2R mRNA was only observed in cells treated with 5 μg/mL LPS and pre-treated with DEX. Finally, a significant effect of DEX co-treatment was observed on MRAP mRNA (Fig. 7D), however, post hoc analysis did not reveal any significant difference between specific treatment groups. We conclude that in the in ATC7-THP1 cells, there is no effect of dexamethasone pre-treatment on LPS induced IL-6 mRNA expression and steroidogenic gene activation with our without subsequent glucocorticoid coadministration.

#### 2.5.7 Effect of LPS on ACTH- induced IL-6 mRNA and steroidogenic gene expression in ATC7 only cells and in ATC7 cells co-cultured with THP1 cells

**Fig. 8:**
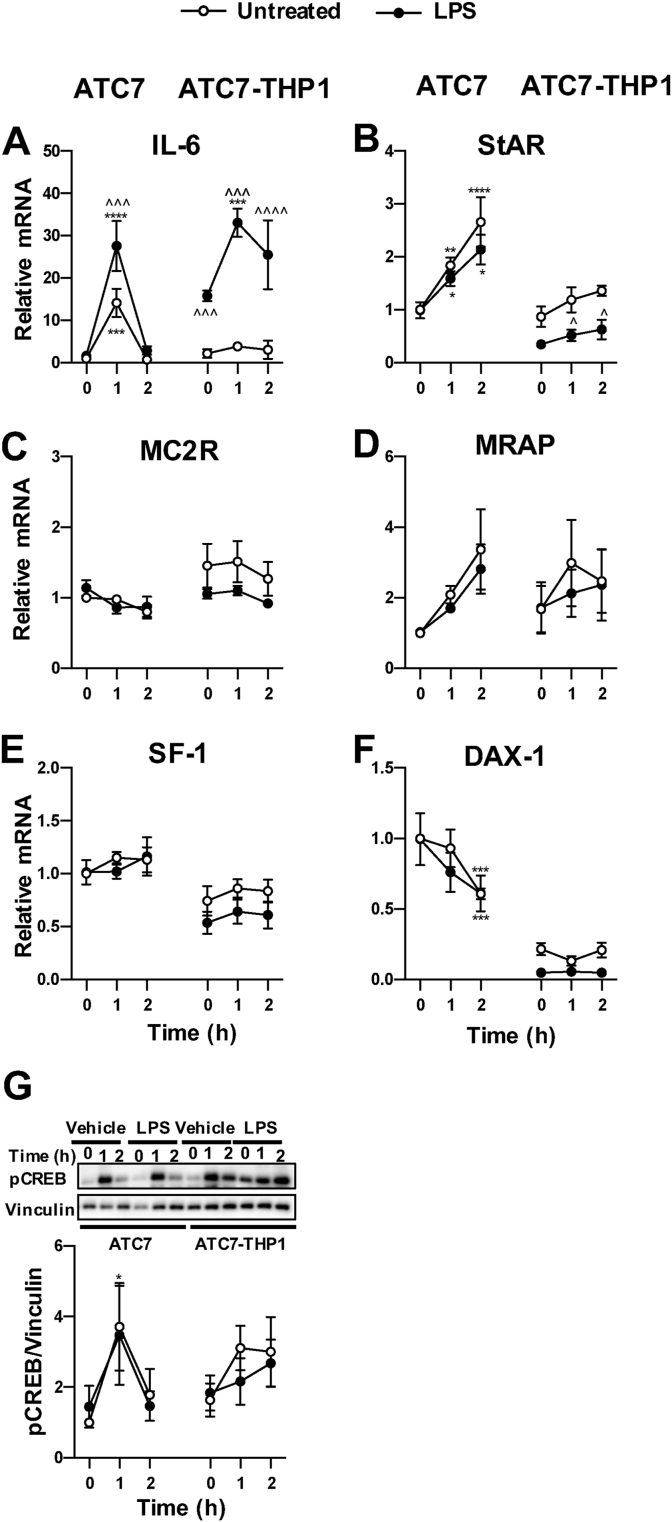
**Effect of LPS on ACTH-induced IL-6 mRNA and steroidogenic pathway activity.** ATC7 cultured alone and ATC7 cells co-cultured with THP1 cells were incubated with LPS (5 μg/mL) and then treated with ACTH for up to 2 h. (**A-F**) IL-6 and steroidogenic genes mRNA levels were measured in ATC7 cells by RTqPCR, and GAPDH was used as a house-keeping gene. (**G**) Relative levels of phosphorylated CREB (pCREB) were measured in ATC7 cells by western immunoblotting, and data were normalized to vinculin. Data are the mean ± SEM (n = 4/group) and are expressed as fold induction of untreated ATC7 cells; data were analyzed by three-way ANOVA followed by Fisher's LSD post hoc test. *P < 0.05; **P < 0.01; ***P < 0.001; ****P < 0.0001 *vs* same treatment ATC7 or ATC7 cells co-cultured with THP1 cells at time 0; ^^P < 0.01; ^^^P < 0.01; ^^^^P < 0.01 *vs* untreated ATC7 or ATC7 cells co-cultured with THP1 cell at the same time-point.

Studies in humans and in rodents have shown that LPS-induced glucocorticoid secretion can occur through its effects on the HPA axis (Chrousos 1995). In addition to regulating the secretion of CRH in the hypothalamus, and of ACTH in the pituitary, LPS administration directly activates the adrenal gland steroidogenic pathway and can potentiate the effects of ACTH on glucocorticoid synthesis (Kanczkowski et al., 2016). Therefore, we decided to investigate the effects of LPS treatment on Il-6 and steroidogenic genes mRNA in both ATC7 alone and ATC7 cells co-cultured with THP1 cells (Fig. 8). In these experiments set, ATC7 only cells and ATC7-THP1 cells were treated with LPS 5 μg/mL for 24 h and then treated with ACTH 10 nM for up to 2 h. Three-way ANOVA analysis of IL-6 mRNA data showed a significant effect of ACTH (P < 0.0001), LPS (P < 0.0001), THP1 (P = 0.02) as well as ACTH x THP1 (P = 0.007), LPS x THP1 (P < 0.0001) and ACTH x LPS (P = 0.01) interactions (Fig. 8A). To our surprise, we found that ACTH alone increased IL-6 mRNA levels in ATC7 cells, and this effect was potentiated by pre-treatment with LPS. Interestingly, ACTH alone did not increase IL-6 mRNA in ATC7-THP1 cells, whereas a significant increase was observed when ATC7-THP1 cells were treated with LPS.

Analysis of StAR mRNA revealed a significant effect of ACTH (P < 0.0001), LPS (P = 0.001) and THP1 (P < 0.0001) as well as a significant ACTH x THP1 interaction (P = 0.005) (Fig. 8B). As expected, StAR mRNA levels were increased in ATC7 only cells treated with ACTH, and LPS did not affect such effect. However, the increase in StAR mRNA induced by ACTH was reduced in ATC7-THP1 cells, an effect that was further potentiated by LPS. A significant effect of ACTH (P < 0.0001) and THP1 (P = 0.02), as well as THP1 x LPS interaction (P = 0.05) was also observed on MC2R mRNA levels (Fig. 8C). However, while there were no significant changes in ATC7 cells treated with ACTH, even following pre-treatment with LPS, MC2R mRNA levels were higher in ATC7-THP1 cells treated with ACTH only, when compared to ATC7 only cells. DAX-1 mRNA levels were also affected by both ACTH (P = 0.03) and THP1 (P < 0.0001), with a significant effect of ACTH x THP1 interaction (P = 0.02), whereas only a trend of the effect of LPS was observed (P = 0.08) (Fig. 8F). DAX-1 mRNA levels were decreased in ATC7 cells treated with ACTH ± LPS at 2 h, compared to time 0, whereas a significant decrease was observed in ACT7-THP1 cells prior to ACTH treatment, and no further decrease was observed after ACTH treatment, nor LPS treatment had any further effect. To our surprise, only a trend of effect of ACTH was observed on MRAP (Fig. 8D), while a significant effect of THP1 (P < 0.0001), and a trend of effect of LPS (P = 0.09), was found on SF-1 mRNA levels, with a significant overall decrease in ATC7-THP1 cells treated with LPS (Fig. 8E). In these experiments, we demonstrate that the LPS and ACTH induced adrenal IL-6 mRNA expression and steroidogenic genes activation are significantly modulated by the THP1 cells.

To evaluate whether the decrease in ACTH-induced StAR mRNA in ATC7 cells co-cultured with THP1 cells was associated with a decreased activation of CREB, we measured the levels of pCREB using Western immunoblot (Fig. 8G). Although there was no significant effect of ACTH, LPS or THP1, a significant ACTH × LPS interaction was detected (P = 0.02). Post hoc analysis revealed that while ACTH increased pCREB levels in ATC7 only cells pre-treated with vehicle (P = 0.02), only a trend of effect was found in ATC7 only cells pre-treated with LPS (P = 0.07), and no significant effect of ACTH was found in ATC7-THP1 cells.

## 3 Discussion

Recent data have provided evidences of HPA axis-independent, intra-adrenal mechanisms involved in the regulation of glucocorticoid release during acute inflammatory stress (Boonen et al., 2015). It is likely that such mechanisms could complement or augment the well-known HPA axis activation during critical illness. The adrenal tissue microenvironment contains a variety of cells, including neural cells, adipocytes, endothelial and immune cells, that could indeed regulate adrenal steroidogenesis (Boonen et al., 2015). The interaction of steroidogenic cells with immune cells is of particular importance because several studies have shown that the generalized inflammation that accompanies acute stress is associated with an infiltration of the adrenal cortex by immune cells (Kanczkowski et al., 2013a; Jennewein et al., 2016). This immune-steroidogenic cross-talk could occur either through the activation of residence macrophages and/or by the recruitment of circulating immune cells into the adrenal cortex. One study suggested that systemic immune cells, rather than the adrenal cells, are the major regulator of the TLR-mediated adrenal activation (Kanczkowski et al., 2013b). It is undoubtedly the case that the adrenals glands, like the thyroid gland, have the highest blood supply *per* gram of tissue in the body, and it is likely that the adrenal tissue will be flooded by immune-effector cells during acute inflammatory stress.

The current study reports the characterization of a novel co-culture model to investigate these interactions. The use of the adrenocortical tumour ATC7 cell line with complete *zona fasciculata* cell phenotype enabled us to assess the effect of an inflammatory stimulus on the expression of the pro-inflammatory cytokine IL-6 mRNA and the expression of key steroidogenic genes. Rat and human adrenal cells do express a variety of pro-inflammatory cytokines in response to immune activation, including TNFα, IL-1, IL-6, IL-18, TGFβ (Judd 1997; Bornstein et al., 2004a). We have chosen to measure the expression of IL-6 because it can be induced by inflammation directly as well as in response to IL-1β. Furthermore, several studies have shown that IL-6 can affect adrenal steroidogenesis either directly or via activation of the CRH-ACTH axis (Bethin et al., 2000; Bornstein et al., 2004a; Chrousos et al., 2015). In humans, the presence of IL-6, IL-6 receptor and IL-6 mRNA in the adrenal cortex suggests that IL6 could play a paracrine or autocrine role in the immune, adrenal cross-talk (Päth *et al.* 1997; Gonzalez-hernandez and Scherbaum, 2016). We decided to use the THP1 cell line because this is a commonly used model to study monocyte/macrophage functions (Tsuchiya et al., 1980). THP1 cells have been used before in other co-culture models including vascular smooth muscle cells (Li et al., 2006; Zhang et al., 2008), adipocytes (Spencer et al., 2010), T-lymphocytes (Azenabor et al., 2011), platelets (Aslam et al., 2007) and intestinal cells (Watanabe et al., 2004). Furthermore, THP1 cells, and particularly the matured macrophages, are known to secrete several pro-inflammatory cytokines as a result of LPS stimulation including TNFα, IL-1β, IL-6, IL-8 and IL-10 (Wehrhahn et al., 2010; Palacio *et al.* 2011; Schildberger et al., 2013b).

In the present study, we show a significant increase in IL-6 mRNA expression in ATC7 cells in response to LPS only when these cells are co-cultured with the THP1 cells, suggesting that the expression of adrenal pro-inflammatory cytokines in response to inflammatory stress is dependent on the presence of immune cells. Because LPS had no effect on ATC7 cells alone, we hypothesize that, in our co-culture experimental model, LPS induces the secretion of cytokines by THP1 macrophages which then acts on the adrenal cells resulting in the expression of IL-6 mRNA. We have also found that the effects of LPS on IL-6 mRNA is dependent on the ATC7 to THP-1 cell ratio. This suggests that *in vivo*, the increased expression of adrenal pro-inflammatory cytokines during acute stress could occur by increased recruitment of immune cells into the adrenal cortex. As discussed above, THP1 cells secrete a number of pro-inflammatory cytokines in response to LPS stimulation, including TNFα, IL-1β, IL-6, and IL-10 (Wehrhahn et al., 2010; Palacio *et al.* 2011; Schildberger et al., 2013b). Wehrhahn et al. investigated the function of the transient receptor potential melastatin 2 (TRPM2) in the LPS induced cytokine production by the THP1 cells at 1 h, 4 h and 16 h. They were able to measure significant increases in TNFα, IL-6, IL-8 and IL-10 (Wehrhahn et al., 2010). Palacio et al. investigated the anti-inflammatory effect of N-acetylcysteine (NAC) on LPS activated THP1 macrophages under mild oxidative conditions. The cytokine mRNA and protein for IL-1 β, TNFα, IL-6, IL-8 and IL-10 were measured in the cell culture supernatants at 2, 4, 6 and 24 h. In the absence of NAC, the TNFα mRNA peaked at 2 h from LPS stimulation and gradually decreased up to 24 h compared to the untreated cells. The IL-1β mRNA was elevated between 2 and 6 h then decreased at the 24-h time point, and the IL-6 mRNA peaked between 4 and 6 h (Palacio *et al.* 2011). Schildberger et al. measured the cytokine concentrations in the cell media (TNFα, IL-6, IL-8 and IL-10) after LPS stimulation of THP1 cell in comparison to the cytokine release pattern of isolated human peripheral blood mononuclear cells (PBMC) and monocytes (Schildberger et al., 2013a). In Schildberger et al. study, TNFα peaked at 4 h, while the IL-1β concentrations peaked at 6 h and remained elevated up to 24 h. They also found the THP1 cells did not secrete any IL-6 and IL-10 in the media after LPS stimulation and secreted far less IL-8 compared to human peripheral blood mononuclear cells (PBMC) and monocytes. However, the THP1 had comparable TNFα secretion to human peripheral blood mononuclear cells (PBMC) and monocytes. In light of these studies, we investigated the time course of the effects of IL-1β, IL-6 and TNFα on IL-6 and steroidogenic gene expression in ATC7 cells alone and in ATC7 cells co-cultured with THP1 cells. Our results show that treatment with cytokines can affect IL-6 mRNA, a result that is consistent with previous studies (Judd and MacLeod 1992), and steroidogenic gene expression, and that these effects are different in ATC7-THP1 and ATC7 alone. Interestingly, we found differences in the dynamics of IL-1β and TNFα effects on IL-6 mRNA, that is, a more rapid response in ATC7 cells alone, and, surprisingly, IL-6 induced a small but significant increase in IL-6 mRNA expression in ATC7 cells cultured alone, but not in co-cultured cells, suggesting that co-incubation with THP1 has protective effect on pro-inflammatory response to IL-6 in the adrenal. Changes in steroidogenic gene expression were also observed in response to cytokines in both ATC7 and ATC7-THP1 cells, including a decrease in StAR mRNA in response to TNFa, which is consistent with the effects observed in ATC7-THP1 cells treated with LPSWe also observed changes in MRAP mRNA, with both a decrease and an increase following IL-1β and IL-6 treatments, respectivelly, and a decrease in DAX-1 mRNA following IL-1β treatment, although such effect only reached stistical significance in the post hot test in co-cultured cells (MRAP mRNA) or in ATC7 cells alone (DAX-1 mRNA). These changes in DAX-1 are also constent wih the effects of LPS treatment in ATC7-THP1 cells. The data are important as they provide an insight of the role of specific cytokines in regulating immune and steroidigenic response in adrenal glands exposed to inflammatory stimulus.

We have also assessed the effect of glucocorticoids on immune-adrenal interactions. This approach is novel since, to our knowledge, the effects of glucocorticoids on the HPA axis responses to inflammation has only been investigated at a system level, and not directly in the adrenal gland cells. We found a significant effect of a high dose of LPS on the increase of IL-6 mRNA expression. This increase was suppressed by low and medium doses of DEX. A similar dose-dependent suppression was noted in the StAR mRNA expression as a result of LPS stimulation. Furthermore, we found a significant effect of DEX on the DAX-1 mRNA response to LPS stimulation, whereas we noted a trend in a decrease of DAX-1 mRNA expression dependent on LPS and DEX dose co-stimulation. Gummow et al. investigated the direct effect of dexamethasone on the steroidogenic gene expression in primary adrenocortical cells, and they found an increase in DAX-1 mRNA expression and a decrease in StAR mRNA expression that was mediated by glucocorticoid receptor activation (Gummow et al., 2006). In our experiments, we did not find any effect of dexamethasone co-incubation on steroidogenic gene expression in ATCH-THP1 cells in the absence of LPS co-stimulation. Nevertheless, our data further support a direct effect of glucocorticoids on the steroidogenic network activity as shown in previous work from our group (Spiga et al., 2020). This suggests that during acute inflammatory stress, systemic administration of glucocorticoids can directly modulate steroidogenesis in an HPA axis-independent manner.

Furthermore, we investigated the temporal relation between the glucocorticoid response and LPS stimulation in regulating the expression of IL-6 and steroidogenic genes. Despite traditional views according to which glucocorticoids are considered uniformly anti-inflammatory, research in the last decade has suggested that glucocorticoids can have a bimodal action: both pro-inflammatory and anti-inflammatory (Sapolsky et al., 2000; Sorrells et al., 2009). This bimodal effect seems to depend on the time of glucocorticoid administration in relation to the inflammatory stress stimulus. A pro-inflammatory effect of glucocorticoids has been demonstrated in immune-competent cell lines (macrophages) (Smyth et al., 2004) and in the central nervous system (hippocampal microglia) (Frank et al., 2007). We investigated whether this effect occurs within the isolated adrenal cells depending on the time of glucocorticoid administration in relation to the inflammatory stress (LPS stimulation). We found that DEX pre-treatment prevented the LPS-induced IL-6 mRNA response when compared to co-treated cells, suggesting that the so-called bimodal effect of steroids (anti- and pro-inflammatory) on IL-6 regulation that has been described in immune and neural cell lines does not apply to adrenal cells, at least within the experimental conditions used in our studies. (Yeager et al., 2004; Horowitz and Zunszain 2015).

Because ACTH plasma levels increase in response to inflammatory stress, we also investigated the effects of ACTH treatment on IL-6 mRNA and steroidogenic genes mRNA in both ATC7 alone and in ATC7 cells co-cultured with THP1 cells. To our surprise, we found that ACTH alone was able to induce IL-6 mRNA in ATC7 cells, and this effect was potentiated by pre-treatment with LPS. Interestingly, the effect of ACTH on IL-6 mRNA was not observed in ATC7 cells co-cultured with THP1 cells in the absence of LPS, suggesting that anti-inflammatory cytokines secreted by THP1 cells in basal conditions may protect the adrenal cells from a non-inflammatory immune activation mediated by ACTH. We have recently shown that ACTH treatment dynamically increases the expression of steroidogenic genes in ATC7 cells (Hazell et al., 2019). Our present data confirmed our previous findings, but also show that the dynamic effect of ACTH is disrupted in ATC7 cells co-cultured with THP1 cells, with a smaller effect on StAR mRNA, which was further decreased by pre-treatment with LPS, and complete suppression of DAX-1 mRNA. These effects were associated with a decrease in pCREB levels, suggesting that the effects of co-culture with THP1 cells may occur at the levels of cAMP/PKA signalling. Interestingly, the effects of ACTH on other steroidogenic genes, including MC2R, MRAP and SF-1 were not affected by co-culture with THP1 cells, nor by pre-treatment with LPS. The effect of ACTH on IL-6 mRNA and steroidogenic genes was significantly different in the presence of THP1 cells. IL-6 mRNA and phosphorylation of CREB appeared enhanced by ACTH in the presence to THP1 cells and LPS, while the suppression of STAR mRNA and DAX-1 mRNA was more pronounced in the LPS-treated cells, compared to vehicle-treated ATC-THP1 cells. A link between an increase in CREB phosphorylation and progesterone levels in response to IL-1β has been shown in *granulosa* cells (Dang et al., 2017). Therefore, it is tempting to speculate that the effects of immune stimulation in adrenocortical cells may occur by a similar mechanism. Overall our results suggest that immune-adrenal cross-talk may be integrated with the hormonal response of the HPA axis during acute stress.

In conclusion, we report a novel co-culture model suitable for assessing immune-adrenal interactions in the context of stress. We demonstrated that the expression of pro-inflammatory adrenal cytokines after LPS stimulation is dependent on the ratio of adrenal and immune cells. We have also noted that the presence of THP1 cells can modulate the response of the steroidogenic gene network to LPS activation, and this is further modulated by ACTH stimulation. Further work is needed to understand the cytokine interaction that occurs between the immune and adrenal cells and its correlation to the steroidogenic gene activation during stress.

## Declaration of interest

The authors have nothing to declare.

## Funding

This work was funded by the Medical Research Council Grant MR/J008893/1( to FS, GHazell and SLL), by the National Institute for Health Research (NIHR) Biomedical Research Centre at University Hospitals Bristol NHS Foundation Trust and the University of Bristol (to DPF, GHorn and GDA), and the British Hearth fundation Grant CH/1992027/7163 (to GDA). The views expressed in this publication are those of the author(s) and not necessarily those of the NHS, the National Institute for Health Research or the Department of Health and Social Care.

## CRediT authorship contribution statement

**Daniel P. Fudulu:** Conceptualization, Methodology, Formal analysis, Investigation, Writing - original draft, Writing - review & editing. **George Horn:** Formal analysis, Investigation, Methodology. **Georgina Hazell:** Methodology. **Anne-Marie Lefrançois-Martinez:** Methodology, Writing - review & editing. **Antoine Martinez:** Methodology, Writing - review & editing. **Gianni D. Angelini:** Conceptualization, Funding acquisition. **Stafford L. Lightman:** Conceptualization, Funding acquisition, Writing - review & editing. **Francesca Spiga:** Conceptualization, Formal analysis, Writing - original draft, Writing - review & editing, Visualization, Supervision, Project administration, Funding acquisition.
