# Supplementary material for: Co-culture of monocytes and zona fasciculata adrenal cells: An in vitro model to study the immune-adrenal cross-talk
Source: Mol Cell Endocrinol. 2021 Apr 15;526:111195. doi: 10.1016/j.mce.2021.111195 (PMC8024787; doi:10.1016/j.mce.2021.111195)
Supplement: Multimedia component 1 [file mmc1.pdf]

Supplementary Table 1: RTqPCR primers sequence

Gene target	Forward	Reverse
GAPDH mRNA	CCATCACTGCCACCCAGAAGA	GACACATTGGGGGTAGGAACA
IL6 mRNA	GCCTTCTTGGGACTGATGCT	GCCATTGCACAACTCTTTTCTCA
StAR mRNA	TCGTGAGCGTGCGCTGTACC	CTTCGGCAGCCACCCCTTCAG
MC2R mRNA	CCAAGGCCCTTCTAAGCCAG	CTTGCGGTGTCATTGGTGTG
MRAP mRNA	AGTCATGGCCAACGGGACCG	GGGACTGTGCCTCATCTGTGGGG
SF-1 mRNA	AGGAGGAAAGGACGATCGGA	ACCTTGTCACCACACATGG
DAX-1 mRNA	ACCGTGCTCTTTAACCCAGA	CCGGATGTGCTCAGTAAGG

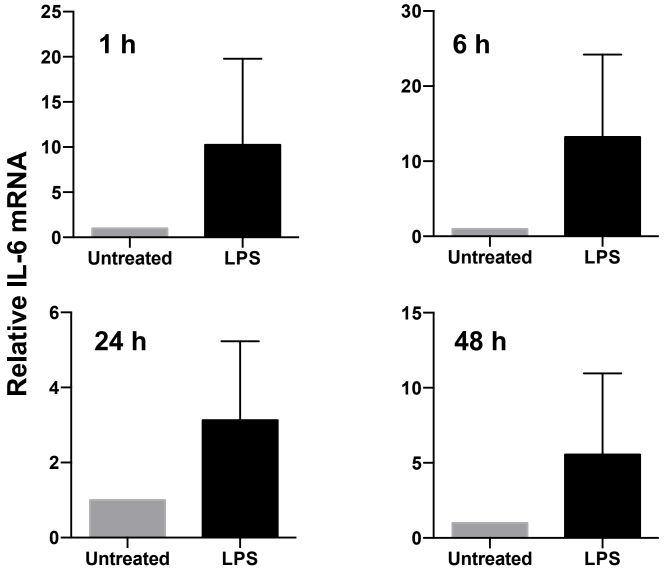

Supplementary Figure 1. Effect of LPS on IL-6 mRNA in ATC7 cells. ACT7 cells were treated with LPS (10 µg/mL) for 1, 3, 6 and 12 h. Relative levels of Il-6 mRNA were measured by RTqPCR and IL-6 mRNA expression was normalised to GAPDH. Data are mean ± SEM of three separate experiments and are expressed as fold induction of untreated ATC7 cells. Data were analysed using unpaired samples Student's t-test.

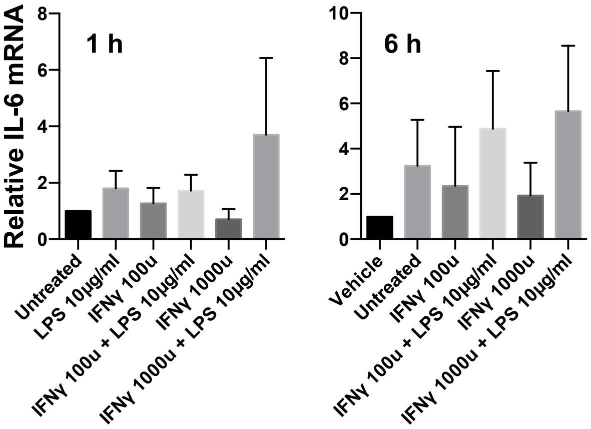

Supplementary Figure 2. Effect of IFNγ and LPS on IL6 mRNA in ATC7 cells. ACT7 cells were treated with LPS (10 µg/mL) and/or IFNγ (100u or 1000u) for 1 and 6 hours. Relative levels of Il-6 mRNA were measured by RTqPCR and the IL-6 expression was normalised to GAPDH. Data are mean ± SEM of three separate experiments and are expressed as fold induction of untreated ATC7 cells. Data were analysed using unpaired samples Student's t-test.
